# Can gene expression profiling predict survival for patients with squamous cell carcinoma of the lung?

**DOI:** 10.1186/1476-4598-3-35

**Published:** 2004-12-03

**Authors:** Zhifu Sun, Ping Yang, Marie-Christine Aubry, Farhad Kosari, Chiaki Endo, Julian Molina, George Vasmatzis

**Affiliations:** 1 Department of Health Sciences Research, Mayo Clinic, Rochester, Minnesota 55905, USA; 2 Division of Anatomic Pathology, Mayo Clinic, Rochester, Minnesota 55905, USA; 3 Laboratory of Bioinformatics and Molecular Biology, Mayo Clinic, Rochester, Minnesota 55905, USA; 4 Division of Medical Oncology, Mayo Clinic, Rochester, Minnesota 55905, USA

## Abstract

**Background:**

Lung cancer remains to be the leading cause of cancer death worldwide. Patients with similar lung cancer may experience quite different clinical outcomes. Reliable molecular prognostic markers are needed to characterize the disparity. In order to identify the genes responsible for the aggressiveness of squamous cell carcinoma of the lung, we applied DNA microarray technology to a case control study. Fifteen patients with surgically treated stage I squamous cell lung cancer were selected. Ten were one-to-one matched on tumour size and grade, age, gender, and smoking status; five died of lung cancer recurrence within 24 months (high-aggressive group), and five survived more than 54 months after surgery (low-aggressive group). Five additional tissues were included as test samples. Unsupervised and supervised approaches were used to explore the relationship among samples and identify differentially expressed genes. We also evaluated the gene markers' accuracy in segregating samples to their respective group. Functional gene networks for the significant genes were retrieved, and their association with survival was tested.

**Results:**

Unsupervised clustering did not group tumours based on survival experience. At p < 0.05, 294 and 246 differentially expressed genes for matched and unmatched analysis respectively were identified between the low and high aggressive groups. Linear discriminant analysis was performed on all samples using the 27 top unique genes, and the results showed an overall accuracy rate of 80%. Tests on the association of 24 gene networks with study outcome showed that 7 were highly correlated with the survival time of the lung cancer patients.

**Conclusion:**

The overall gene expression pattern between the high and low aggressive squamous cell carcinomas of the lung did not differ significantly with the control of confounding factors. A small subset of genes or genes in specific pathways may be responsible for the aggressive nature of a tumour and could potentially serve as panels of prognostic markers for stage I squamous cell lung cancer.

## Background

Lung cancer remains to be the leading cause of cancer death in many European and North American countries [1,2]. It accounts for 13% of all cancer diagnoses but is responsible for nearly 30% cancer deaths in the United States [2]. Substantial effort has been made to identify prognostic factors that can be used for better patient management and improved survival. As of 2001, as many as 169 prognostic factors were identified in Non-Small Cell Lung Cancer (NSCLC) [3]. However, only very few such as TNM stage or patient performance status are consistent predictors, but they still can not predict individuals' prognosis accurately within a stage. Indeed, why do some patients with stage I lung cancer progress very quickly while others survive for a long time cancer free? This puzzle naturally has prompted researchers to contemplate whether the aggressive nature of NSCLC is genetically predetermined and whether the difference in gene expression could be identified as a more reliable clinical outcome predictor.

Searching for molecular prognostic markers is traditionally carried out by analyzing one or several gene expression products at a time, which can only touch a very small fraction of expressed genes in the genome. Fortunately, recently developed high-throughput technologies such as DNA microarray provide promising and efficient screening tools for this purpose. It has been used in lung cancer research to identify the subclasses associated with tumour differentiation and patient survival [4,5], to predict patient survival or potential metastasis of a tumour based on gene expression profiles [6-8], and to compare two predefined classes such as tumour vs. normal or smokers vs. non-smokers to reveal differentially expressed genes [9-13]. However, some of these findings are simply a reiteration of diagnoses that can be easily made by standard pathologic evaluation, and their added clinical values are limited. In addition, two major issues exist in most of those studies to search for prognostic markers: (1) Case selection criteria were not clearly defined. Different tumour type, grade, stage, treatment, and smoking history were often mixed together, making it difficult to assess whether gene expression profiling discriminated patient survival independent of other known predictors. Although tumour type and grade of differentiation are not consistently documented as prognostic factors, they are very important in determining a sample's class membership in gene expression profiling [4-6]. (2) A clustering approach has been used as a major analytical tool to characterize cancer phenotypes including histological type, metastatic potential or patient survival. However, clustering is more appropriate to visualize gene expression patterns, and its results are heavily affected by the distance matrix and linkage method selected [14]. The existing evidence supports the notion that a clustering algorithm mainly groups samples based on histology, a variable not yet proven as an independent factor in NSCLC prognosis. This reemphasizes a central question of whether a clustering approach can discern the aggressive nature of a tumour with the same histological type.

In order to answer the question why do some patients with stage I squamous cell carcinoma progress rapidly after curative resection while others survive a long time without disease recurrence, we designed a case control study matching on important prognostic factors so that only the tumour genetic factor was assumed to be a major determinant in patients' prognosis. We explored whether the widely-used hierarchical clustering was applicable in our study and whether the differentially expressed genes or functionally related groups of genes had any predictive value in an independent group of similar patients.

## Results

### Clinical Characteristics of Selected Patients

The clinical characteristics of the 15 stage I squamous cell carcinoma patients in our study is provided in the Additional file 1. Since the first ten samples were matched and used for the initial marker selection, the two groups (sample# 1–5 vs. 6–10) were well balanced in terms of age, gender, tumour size, smoking history, and treatment. The characteristics of the additional five test samples were very similar to the group of low aggressive samples.

### Unsupervised Clustering

**Figure 1:**
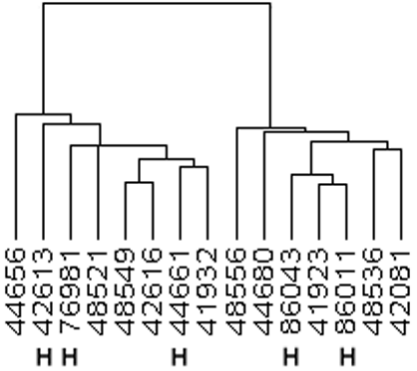
**Hierarchical clustering for 15 samples. **2810 probe sets filtered by: standard deviation/mean across all samples > 0.06; and the expression level on the log2 scale ≥ 4.00 in ≥ 60% of the samples. H: indicates high aggressive tumors.

When a subset of 2810 filtered genes was used to conduct hierarchical clustering for all 15 samples, two main clusters were formed (Figure 1). However, the clusters did not distinguish the two groups by survival outcome: high-aggressive and low-aggressive tumours were almost evenly distributed within each cluster. Three of the high-aggressive tumours were present in the left cluster and two in the right. For the ten low-aggressive tumours, five were in the left cluster and five were in the right.

### Class Comparison and Top Candidate Gene Selection

**Figure 2:**
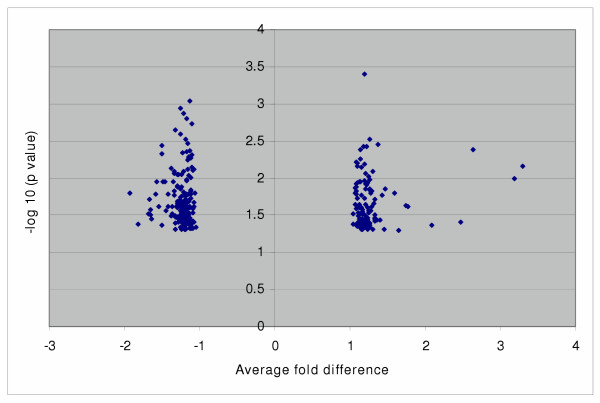
**Distribution of significant genes from matched analysis. **294 significant genes (P < 0.05) selected by matched analysis are plotted by fold difference (x-axis) vs. p value using t-test (y-axis) A y-axis greater than 1.3 is equivalent to a p value less than 0.05, and greater than 2 is equivalent to a p value less than 0.01. A positive or negative value at the x-axis indicates genes are up or down regulated in the high-aggressive group compared to the low aggressive group.

**Figure 3:**
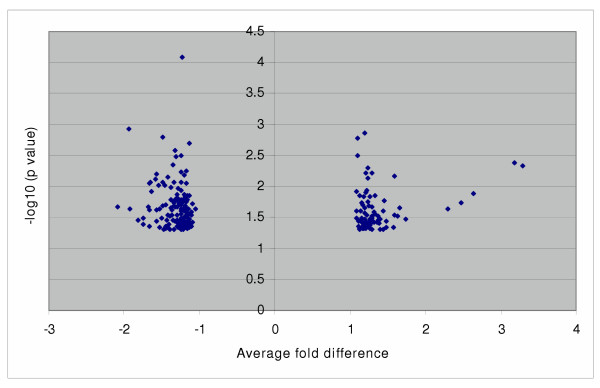
**Distribution of significant genes from unmatched analysis. **246 significant genes (p < 0.05) selected by unmatched analysis are plotted by fold difference (x-axis) vs. p value using t-test (y-axis) A y-axis greater than 1.3 is equivalent to a p value less than 0.05, and greater than 2 is equivalent to a p value less than 0.01. A positive or negative value at the x-axis indicates genes are up or down regulated in the high-aggressive group compared to the low aggressive group.

To identify a panel of genes that are differentially expressed between the high and low aggressive groups as potential prognostic biomarkers, we applied matched (pair of 1–6, 2–7, 3–8, 4–9, 5–10) and unmatched (group 1–5 vs. 6–10) t statistics to the ten well-matched samples. At p < 0.05, 294 and 246 genes were significant in matched and unmatched comparison, respectively, with 126 selected by both. The majority of significant genes were within a two-fold mean difference between the two comparison groups with p values ranging from 0.05 to 0.01 (Figure 2, 3).

**Figure 4:**
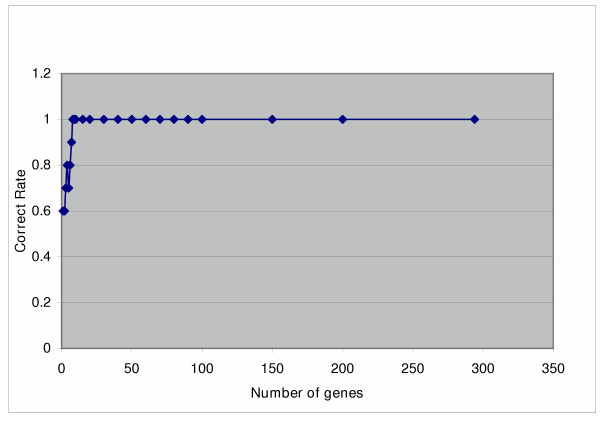
**Leave-one-out prediction on training samples. **The x-axis represents different numbers of significant genes from matched analysis that was used to predict a membership of a sample by the leave-one-out algorithm. The y-axis shows the correct prediction rate for the 10 training samples.

From the list generated by matched analysis, we used 1–10, 15, 20, 30, 40, 50, 60, 70, 80, 90, 100, 150, 200, 250, and 294 genes each time and evaluated their discriminating capability for training samples by leave-one-out algorithm as illustrated in Figure 4. As few as 10 genes were found sufficient to achieve 100% accuracy. The same procedure was performed for the gene list generated by unmatched analysis and similar results were obtained. The first 20 genes from each procedure, which had the highest signal-to-noise ratio and therefore accurately distinguished the training samples with contrasting outcome, were selected and combined. Table 1 lists the 27 unique genes between the two procedures. (There were 2 selected probe sets for each gene *ATP1B1 *and *IGFBP3*, and they were counted once.)

**Table 1:** The top 27 unique genes with highest signal-to-noise ratios

	**Gene symbol**	**Gene name**	**Unmatched**	**Matched**
**Up-regulated genes**	*ATP1B1*	ATPase, Na+/K+ transporting, beta 1 polypeptide		
	*TP53*	tumor protein p53 (Li-Fraumeni syndrome)		
	*CYP26A1*	cytochrome P450, family 26, subfamily A, polypeptide 1		
	*SYCP2*	synaptonemal complex protein 2		
	*IGFBP3*	insulin-like growth factor binding protein 3		
	*CPOX*	coproporphyrinogen oxidase (coproporphyria, harderoporphyria)		
	*MAGEA1*	melanoma antigen, family A, 1 (directs expression of antigen MZ2-E)		
	*H1F0*	H1 histone family, member 0		
	*MAGEA12*	melanoma antigen, family A, 12		
	*	Homo sapiens clone 23705 mRNA sequence		
	*	Homo sapiens cDNA: FLJ21672 fis, clone COL09025.		
	*FLJ20477*	Homo sapiens cDNA FLJ39734 fis, clone SMINT2016146.		

**Down-regulated genes**	*P2RY5*	purinergic receptor P2Y, G-protein coupled, 5		
	*DKFZp586G0123*	hypothetical protein DKFZp586G0123		
	*EPB41L3*	erythrocyte membrane protein band 4.1-like 3		
	*DKFZP586A0522*	DKFZP586A0522 protein		
	*CSAD*	cysteine sulfinic acid decarboxylase		
	*BRIP1*	BRCA1-interacting protein 1		
	*MYC*	v-myc myelocytomatosis viral oncogene homolog (avian)		
	*PDCD4*	programmed cell death 4 (neoplastic transformation inhibitor)		
	*TAF6L*	TAF6-like RNA polymerase II, p300/CBP-associated factor (PCAF)-associated factor, 65 kDa		
	*ABCA12*	ATP-binding cassette, sub-family A (ABC1), member 12		
	*ZNF198*	zinc finger protein 198		
	*NOTCH2*	Notch homolog 2 (Drosophila)		
	*TncRNA*	Human clone 137308 mRNA, partial cds.		
	*CLK1*	CDC-like kinase 1		
	*POGZ*	pogo transposable element with ZNF domain		

Significant in that particular analysis (matched or unmatched)

* No gene symbol for these genes

### Linear Discrimination Analysis

We applied linear discrimination analysis to the 27 genes selected from the previous step to assess the accuracy of class membership prediction for both the training and the test samples (Table 2). The overall error rate was 20% (3/15). Interestingly, the linear discrimination score of incorrectly classified samples was among the lowest (absolute value), suggesting a borderline expression pattern between the high and low aggressive tumours.

**Table 2:** LDA classification using 27 top genes

**Sample**	**LD score**	**Class**	**Prediction**	**Probability**	**Correct?**
48521	-0.52	1	1	0.75	Yes
48536	-0.94	1	1	0.87	Yes
41923	-0.26	1	1	0.63	Yes
48549	-0.52	1	1	0.74	Yes
44680	-2.9	1	1	1	Yes
42613	1.0	2	2	0.89	Yes
76981	0.52	2	2	0.75	Yes
44661	2.08	2	2	0.99	Yes
86043	-0.19	2	1	0.59	No
86011	1.71	2	2	0.97	Yes
42616	0.12	?	2	0.56	No
48556	0.05	?	2	0.52	No
41932	-0.88	?	1	0.86	Yes
42081	-0.52	?	1	0.75	Yes
44656	-0.08	?	1	0.54	Yes

LD score: Linear discrimination function calculated value for a given sample; Class: a sample's membership to the low aggressive group (1) or high aggressive group (2), "?" is a test sample whose membership is not known for the procedure and needs to be predicted. Prediction: predicted sample membership. Probability: probability of a sample belonging to a given class based on the classifiers. "Correct?": Whether the prediction is correct compared to the true class of a sample.

### Gene Network Analysis

The test statistics for all genes (22215) using R-package "global test" was not significant, indicating that the overall pattern was similar between high-aggressive and low aggressive groups in our study sample. Using the 126 overlapped genes between matched and unmatched comparisons, we found 24 gene networks from Ingenuity Pathways Knowledge Base. We performed an association test on each network and found that seven were strongly associated with survival (Table 3). We then used the genes in each of the seven networks to predict all 15 samples separately and detected an error rate ranging from 0 to 47%, with *RAB6A *network genes predicting all samples correctly.

**Table 3:** Gene networks associated with survival

**Network genes**†	**Score**‡	**Association test p**	**Prediction error**
***APLP2***, *ARL6IP*, *CASP3*, *CCNG1*, ***CSF1***, *DNMT1*, *EPHA2*, *ERCC3*, ***ERCC5***, *F*2, ***F5***, ***FGF2***, *FUBP1*, *GPI*, *HAS2*, *HMOX2*, ***IGFBP3***, ***LOC283120***, *LOC91768*, *MDM4*, ***MYC***, *P53AIP1*, *PCNA*, *PEG3*, ***RARB***, ***RPL21***, ***RPS6***, *RRM2B*, *TAGLN2*, *THBD*, *TMSB4X*, ***TP53***, *TP53I3*, *TP73*, *WT1*	17	0.01*	7/15
*AMSH*, *AR*, *ATF2*, *BAG1*, *BCL2L1*, *CREBBP*, ***CYBA***, *ENO1*, *EP300*, *FOXG1B*, *HOXA9*, *HOXC8*, ***HSF2***, ***MADH1***, *MADH2*, *MADH3*, *MADH4*, *NCF1*, *NCF2*, *RBM14*, ***RNF14***, ***RTN1***, *RUNX2*, ***SIAH1***, *SP3*, *TOB1*, ***TP53***, *UBE2E3*, *UBE2I*, *ZNF8*	9	0.02*	3/15
***SAC***, *TEC*	2	0.01*	4/15
*RIPK1*, ***TRIAD3***	2	0.03*	4/15
*MIR*, ***TMEM4***	2	0.01*	3/15
*NSF*, *PIK3CG*, ***RAB6A***, *RAB6KIFL*	2	0.01*	0/15
*ATP12A*, *ATP1A1*, *ATP1A2*, *ATP1A3*, ***ATP1B1***, *FXYD7*	1	0.004*	3/15

† Genes in bold face are focus genes (among 126 genes submitted to the Ingenuity knowledge base). ‡ Score indicates the probability that a collection of focus genes could be found in a given network by chance. It is the negative logarithm of the possibility. A score of 2 indicates that chance is only 1%. * Indicates a strong association between the expressions of genes in a network and survival.

## Discussion

To address the critical clinical question of whether the aggressive nature of squamous cell carcinoma of the lung is genetically pre-programmed, we conducted a matched experiment using DNA microarray. The purpose of the design is to control for known confounding factors so that the true association between gene expression and patient survival can be determined. Our results have shown that microarray technology provides both opportunities and challenges in the identification of potential molecular prognostic markers.

In our study, unsupervised clustering did not accurately separate patients based on their clinical outcome behavior. This is in contrast to some investigators [4,5] who, using a similar approach, have identified subclasses of tumours showing differing gene expression profiles correlated with varied clinical outcomes. Using the 19.2 K cDNA microarray chip, Wigle et al [7] successfully partitioned 39 mixed histological types and stages of non-small cell lung cancer into two distinct clusters, those with early recurrence and those without recurrence regardless of tumour types. There are several possible explanations for discrepant results between the studies. First, the formation of clusters is heavily affected by the number of genes used for clustering, the gene selection method, and the clustering algorithm. Highly varied genes generally dominate the clustering process and thus explain why highly different groups such as among subtypes of non-small cell lung cancer (squamous cell carcinoma vs. adenocarcinoma), primary vs. metastatic cancer, or cancer vs. normal tissue, can be reliably differentiated using this technique. However, for the same primary tumour where the clinical outcome is the only noticeable difference, as in our study, this approach might not be as useful. Second, it is not clear in Bhattacharjee et al and Garber et al studies [4,5] whether the gene expression profile was influenced by other prognostic factors such as stage, or whether it was truly a specific and an independent prognostic factor. Finally, differences in tumour series, microarray chip platforms, or data pre-processing could affect results across studies, even within a study [15].

In searching for genes responsible for tumour behavior and patient survival, a case control comparison between two different clinical outcomes (long survival vs. short survival or disease free vs. quick recurrence) or a survival cohort using Cox's proportional hazards model to find gene-outcome association are among the most common options [6,7,9,13]. However, a careful design and implementation for this type of study needs to be taken into consideration since a case-control or survival cohort design is prone to selection bias, i.e., patients enrolled in comparison groups are different other than the factors under study, which makes them incomparable [16]. Without taking any strategy such as randomization, matching, or stratification to deal with the potential biases, the study results should be reviewed with skepticism [16]. Specifically for microarray study of lung cancer outcome, there are many tumour, host, and environmental related factors that are associated with patient prognosis. The imbalance of these factors between the two comparison groups such as the extent of disease (stage), the presence of other diseases, and treatment makes it difficult to establish the true association. Although results have not been consistent in reporting tumour histology of NSCLC as an independent prognostic factor, available evidence has indicated that it could be important in gene profiling as major histological types could be easily separated by the clustering approach [4-6]. If we do not take histology into consideration in case selection and comparison, a distorted result might occur. In contrast, we focused on one subtype of NSCLC within the same stage and matched on all potential confounding factors of survival. The results showed little overall difference in the gene expression profile between the two outcome groups. Less than 300 probe sets were significant at p < 0.05 from over 22,000 probe sets and 20–30 of them were greater than 1.5 fold change between the two groups, which were hardly separable from random noise.

It is a big challenge for microarray analysis to identify reliable genomic prognostic marker panels that can be generalized to independent samples. In our study, the markers based on signal-to-noise ratio did not perform very well on the independent samples although the small sample size could be partly responsible. The result may suggest: (1) survival of patients with squamous cell carcinoma could be the result of genetic and non-genetic factors acting together. Gene expression difference is only a partial explanation. (2) Gene expression among tumours is very heterogeneous, even for the same histological type. By examining our series of tumours, we noticed that even though cell type and grade were matched, there were still some other variables hard to control for, such as cancer cell growth patterns or the constitutions of cancer stroma. Different amounts of lymphocytes or fibroblasts may contribute to the heterogeneous gene expressions. (3) The aggressive nature of a tumour may be determined by a small portion of cells that acquire metastatic capacity through somatic mutation [17], and it is hard to capture these cells since microarray analysis can only examine a very limited portion of a tumour. (4) The genes responsible for tumour aggressiveness may be part of one or multiple pathways. The genes within specific pathways may not be the most differentially expressed and may be often overwhelmed by background noises across tumours; however, as a functional group, they could potentially determine the behavior of a tumour.

We evaluated the pathway hypothesis by finding related genes in specific gene networks using our candidate genes and tested the correlation between the genes and prognosis. Using this strategy, we identified seven gene networks strongly associated with squamous cell carcinoma survival. Although the functional explanation of an entire gene network to survival is yet to be determined, the association of some individual genes such as *p53, c-myc*, and *PDCD4 *(programmed cell death 4) with lung cancer survival has been well-documented in the literature [18-22]. *Rab6A *and related genes, the network accurately separated tumours with 100% accuracy in our study, are involved in intracellular transport. Whether they are functionally relevant to cancer aggressiveness or just surrogate markers of the true underlying mechanism needs to be further clarified.

Although using carefully-matched samples could potentially unveil a true association, the subjects eligible for inclusion are dramatically reduced, often leading to a relatively small sample size and insufficient power to detect a minor difference or overcome randomness [15]. Facing the reality of low reproducibility using microarray technology, it is important that an experiment starts with a good design to minimize various biases [15]. If results from a well-controlled study are promising, a larger scaled follow-up investigation will be warranted.

## Conclusions

We found that the overall gene expression pattern between the high and low aggressive squamous cell carcinomas of the lung was similar after controlling for confounding factors. However, our results suggest a difference between high and low aggressive cancers may be due to a small number of functionally related genes; these are so-called pathway genes that are often overlooked by commonly used analytical approaches. Whether pathway genes work collectively as more reliable prognostic markers or not needs to be further investigated by more studies with a large number of samples.

## Methods

### Study Design and Sample Selection

Cases were defined as the patients who survived less than 24 months after surgery (high-aggressive group) and controls were those who survived more than 54 months after surgery (low-aggressive group). The patient population, from which the cases and controls were drawn, was comprised of patients diagnosed with lung cancer from 1997 to 2001 who underwent curative resection at Mayo Clinic, Minnesota, USA. These patients were prospectively enrolled and had been actively followed since their initial surgery [23]. We restricted this study to stage I squamous cell carcinoma to gain more homogeneity in morphology and to be focused on a common type of lung cancer. Each case was matched to a control by tumour size and grade, age, gender, and smoking status so that the potential confounding factors could be minimized. For each potential patient, we carefully reviewed their medical records and follow-up data to confirm their clinical outcome and the cause of death if the patients were deceased. From a pool of 304 patients with stage I squamous cell carcinoma, five well-matched pairs were finally selected (See Additional file 1) and used for most of the analyses. Five additional patients who survived more than 52 months were included as a test group (See Additional file 1).

All enrolled patients and use of their tissue samples in the study were approved by our Institutional Review Board. The resected tumour and adjacent lung tissues were fast frozen in -80°C within 30 minutes after the tissues were surgically removed.

### RNA Extraction and Microarray Hybridization

All tissue specimens were reviewed by a pulmonary pathologist (MCA) to confirm their diagnosis and ensure that the tissue was appropriate for the experiments. Specifically, the percentage of total tumour, tumour necrosis, amount of inflammation associated with tumour, and cellularity of stroma were evaluated. In the frozen tissue blocks containing cancer, the non-neoplastic tissue was manually cut away from the block to assure at least 80% of the cancer component. Thirty mm^3 ^of each tissue were sectioned at 20 or 35 μm, collected in a buffer RLT (Qiagen, Valencia, CA) supplemented with β-mercaptoethanol, and homogenized using PT 1200C (Kinematica AG, Luzern, Switzerland) rotor/stator. The total RNA was isolated using the RNeasy kit (Qiagen, Valencia, CA) following the manufacturer's specifications. Microarray experiments were performed at the Mayo Clinic Microarray Core Facility. The quality and quantity of RNA samples were controlled by spectrophotometry and the Agilent 2100 Bioanalyzer. Hybridization washes and scanning were performed following the manufacturer's protocols (Affymetrix, Santa Clara, CA). The HG-U133A chip from Affymetrix was used and contains 22,283 probe sets, which we conveniently refer to as genes in this paper.

### Data Processing and Analysis

The Affymetrix Microarray Analysis Suite version 5 (MAS5) was used to process the scanned chip images. This software generates a cell intensity file for each chip, which contains a single intensity value for each probe cell (cel file). Dchip 1.3 [24,25]() was used to calculate the Model Based Expression Index (MBEI). All chips were normalized against an array with a median overall intensity using the invariant set method, and their images were visually inspected for potential problems prior to any data processing and analysis. The MBEI was calculated using the Perfect Match (PM) only model with outlier detection and correction. The calculated expressions were log2 transformed. Control probe sets were excluded in the down-stream analyses.

As a first step, we employed hierarchical clustering to evaluate the similarity and disparity in overall expression patterns among all 15 samples using a subset of 2810 genes, which were filtered by the following criteria: the standard deviation/mean across all samples >0.06 and the expression level on the log2 scale ≥ 4 in at least 60% of the samples. The distance matrix applied in the clustering was one minus the Pearson correlation coefficient (1-r), which measures the closeness between genes or samples, and the linkage method was centroid, which uses the centers of newly formed clusters (genes or samples) to calculate the distance between clusters [26,27].

In order to detect differentially expressed genes between the high and low aggressive groups, we conducted both matched and unmatched t statistics for the ten matched samples at the criteria of p < 0.05 and at least one present call in each comparison group. Next, we applied a feature selection process to isolate a subset of genes selected from the previous step that had a high discriminate power in separating the two distinct groups of tumours. In each step, one sample was withheld as a test sample, and a signal-to-noise ratio as described by Ramaswamy and colleagues [28] was calculated for each gene using the remaining nine samples in the two groups. Based on the number of genes (features) specified, the procedure chose the top genes with highest signal-to-noise ratios and created a linear model to predict the membership of the withheld sample using a weighted-voting algorithm [28]. This process was repeated 10 times (10 samples), and an error prediction rate was obtained for the specified number of genes. By trying out different numbers of genes, a zero error rate for training samples could be achieved. The minimum number of genes obtaining the zero error rate was chosen as the best candidates.

Linear Discrimination Analysis [29] was applied using the subset of genes selected from the previous step to assess whether the genes can discriminate the high from the low aggressive nature of the training and test samples. This method utilizes all input genes (independent variables) to create a discriminant function that maximizes the ratio of between-group variance and within-group variance so that different classes (dependent variable, either low or high aggressive group in our study) can be better separated. Implicitly, each gene is assigned a weight in the function depending on how a gene separates in the two groups and how this gene correlates with other genes. After computation, each sample was given a discriminant score, predicted class membership, and probability for the assigned class. The prediction rate was calculated to evaluate the performance of the classifiers.

Because of stringent matching criteria, we did not expect dramatic difference between the two comparison groups, as reported by other investigators who did not match comparison groups closely. We hypothesized that genes in certain pathways might play a role in squamous cell carcinoma prognosis. We submitted the significant genes selected by both matched and unmatched analysis to the Ingenuity Pathways Analysis application ()and generated gene interaction networks. A test statistic on the association of gene members in a network with a clinical outcome was carried out by using the R package "global test" [30]. If a small p value (<0.05), particularly permutated when sample size is small, is obtained, there is a strong indication that the group of genes, no matter whether they are up or down regulated in the network, is associated with clinical outcome, i.e., long or short survival in our study.

## Authors' contributions

ZS carried out the data analysis and participated in drafting the manuscript. PY designed the study, oversaw the analysis and interpretation, and participated in writing the manuscript. MC analyzed the tissue samples and contributed to the development of the manuscript. FK prepared the tissue samples for microarray assay. CE contributed to the development of the manuscript. JM contributed to the development of the manuscript. GM contributed to analysis of the data.

## Supplementary Material

Additional File 1 Clinical characteristics of 15 cases of stage I Squamous Cell Carcinoma of the Lung. Click here for file
