# Supplementary material for: Can gene expression profiling predict survival for patients with squamous cell carcinoma of the lung?
Source: Mol Cancer. 2004 Dec 3;3:35. doi: 10.1186/1476-4598-3-35 (PMC544571; doi:10.1186/1476-4598-3-35)
Supplement: Additional File 1 — Clinical characteristics of 15 cases of stage I Squamous Cell Carcinoma of the Lung. [file 1476-4598-3-35-S1.doc]

## Additional file 1

## Clinical characteristics of 15 cases of stage I Squamous Cell Carcinoma of Lung

| **Group** | **Sample**  **Number** | **Tissue ID** | **Age** | **Gender** | **Smoking status**† | **Tumor size (cm)** | **Surgery** | **Time to recurrence*** | **Survival Time*** | **Death** | **Cause of death** |
| --- | --- | --- | --- | --- | --- | --- | --- | --- | --- | --- | --- |
| **Cases** | 1 | 42613 | 73 | M | PS | 5x5x4.5 | lobectomy | 10 | 21 | Yes | Metastasis |
|  | 2 | 76981 | 76 | M | PS | 4x4x4 | lobectomy | 8 | 21 | Yes | Metastasis |
|  | 3 | 44661 | 64 | M | PS | 3.5x3x3 | lobectomy | 5 | 9 | Yes | Metastasis |
|  | 4 | 86043 | 59 | F | PS | <3 | lobectomy | 10 | 11 | Yes | Metastasis |
|  | 5 | 86011 | 78 | F | PS | 2X1.8x1.8 | wedge | 16 | 21 | Yes | Metastasis |
|  |  |  |  |  |  |  |  |  |  |  |  |
| **Controls** | 6 | 48521 | 70 | M | PS | 4.7x3.5x2.5 | lobectomy | No | 62 | No | NA |
|  | 7 | 48536 | 80 | M | PS | 4x4x2 | lobectomy | No | 61 | No | NA |
|  | 8 | 41923 | 65 | M | PS | 3x2.5x2.5 | lobectomy | No | 61 | No | NA |
|  | 9 | 48549 | 62 | F | PS | 3x2.5x2.5 | lobectomy | No | 54 | No | NA |
|  | 10 | 44680 | 70 | F | PS | 2.5x2.5x2 | lobectomy | No | 55 | No | NA |
|  |  |  |  |  |  |  |  |  |  |  |  |
| **Test Samples** | 11 | 42616 | 63 | M | PS | 2x2x1.5 | lobectomy | No | 66 | No | NA |
|  | 12 | 48556 | 78 | M | PS | 4.5x4x3.5 | lobectomy | No | 66 | No | NA |
|  | 13 | 41932 | 62 | F | PS | 3.5x2.5x2.5 | lobectomy | No | 52 | No | NA |
|  | 14 | 42081 | 73 | F | CS | 3.5x3.5x2 | wedge | No | 66 | No | NA |
|  | 15 | 44656 | 76 | F | CS | 5x4.7x3 | lobectomy | No | 59 | No | NA |

† PS, past smoker, CS, current smoker; * Time in months from surgery to the event of death or recurrence or last follow-up; case 6 matched to case 1, 7 to 2, …and 10 to 5.
